# Transcranial Magnetic Stimulation for the Treatment of Pharmacoresistant Nondelusional Auditory Verbal Hallucinations in Dementia

**DOI:** 10.1155/2013/930304

**Published:** 2013-10-02

**Authors:** Anna Marras, Stefano Pallanti

**Affiliations:** ^1^University of Florence, 50137 Florence, Italy; ^2^Institute of Neuroscience, 50121 Florence, Italy; ^3^University of Florence School of Medicine, 50134 Florence, Italy; ^4^Albert Einstein College of Medicine and Montefiore Medical Center, NY 10461, USA

## Abstract

Auditory verbal hallucinations (AVHs) are known as a core symptom of schizophrenia, but also occur in a number of other conditions, not least in neurodegenerative disorders such as dementia. In the last decades, Transcranial Magnetic Stimulation (TMS) emerged as a valuable therapeutic approach towards several neurological and psychiatric diseases, including AVHs. Herein we report a case of a seventy-six-years-old woman with vascular-degenerative brain disease, complaining of threatening AVHs. The patient was treated with a high-frequency temporoparietal (T3P3) rTMS protocol for fifteen days. A considerable reduction of AVHs in frequency and content (no more threatening) was observed. Although further research is needed, this seems an encouraging result.

## 1. Introduction

Transcranial Magnetic Stimulation (TMS) is a noninvasive technique of neuromodulation and neurostimulation based on the principle of electromagnetic induction of an electric field in the brain. It was originally developed for the of study brain function [1]. Since Barker and colleagues first demonstrated TMS, it has been widely used earlier to investigate motor conduction and control, later in the study of cognitive functions and brain plasticity. TMS is widely studied in the diagnosis and treatment of neurological and psychiatric diseases, too.

A hallucination is often referred to as “a sensory experience, which occurs in the absence of corresponding external stimulation of the relevant sensory organ, has a sufficient sense of reality to resemble a veridical perception, over which the subject does not feel s/he has direct and voluntary control and which occurs in the awake state” [2]. Auditory verbal hallucinations (AVHs) are defined as perceptions of speech without actual auditory stimulation and represent one of the most common hallucinatory experience, which are a characteristic symptom of schizophrenia [3] but also occur in a wealth of conditions, both neurological/neurodegenerative (e.g., epilepsy, Alzheimer's disease, Parkinson's disease, dementia, and hearing impairment) or psychiatric (e.g., dissociative disorders, mood disorders, and borderline personality disorder).

## 2. TMS and Auditory Hallucinations

Hoffman and colleagues [4] published the first report demonstrating a reduction in auditory verbal hallucinations (AVHs) after a low-frequency (1 Hz) rTMS treatment to the posterior superior temporal gyrus (STG). This brain area and, overall, the temporoparietal cortex (TPC) are involved in speech perception [5, 6] and are active during auditory hallucinations [7–9]. After the publication by Hoffman and colleagues, a number of studies replicated the positive findings using this rTMS protocol (for a complete review see Table 1), and, additionally, several meta-analyses supported the usefulness of rTMS in the treatment of hallucinations [10–12]. Most studies examined AVHs in schizophrenia, while there's a lack of studies about TMS to treat hallucinations in dementia, even though a number of studies reported the presence of psychotic symptoms (mostly delusions and hallucinations) in Alzheimer's and Parkinson's diseases (for a more detailed review see [13, 14]).

## 3. RDOCs and Circuitries Involved

**Figure 1:**
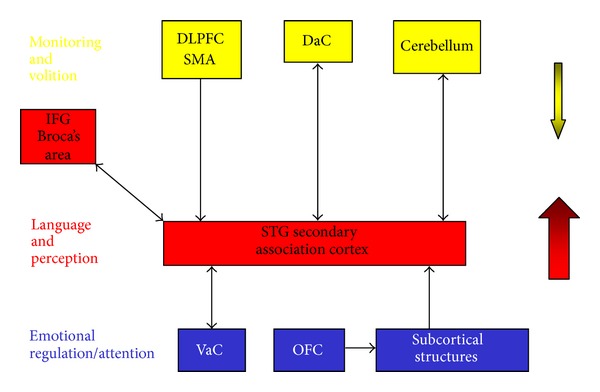
A neuroanatomical model of the hallucinating brain [19].

Though the exact pathophysiological mechanisms of AVHs remain actually unclear, neuroimaging studies have shown abnormalities in a distributed network of brain regions, providing valuable information about brain activity either during hallucinations or at rest. Studies on AVHs mainly employ neurophysiological methods (i.e., EEG), functional magnetic resonance imaging (fMRI), and, recently, transcranial magnetic stimulation (TMS). Most EEG studies showed changes in activity in the left superior temporal gyrus [15–18], not surprisingly since the involvement of this area in auditory processing. fMRI studies contributed much to understanding brain activity in AVHs: Allen and colleagues [19] proposed a neuroanatomical model of hallucinations including brain areas involved in linguistic processing, attention, memory, and emotion. In particular regarding AVHs, they hypothesized an altered connectivity between the superior temporal, inferior frontal gyrus and anterior cingulate cortex that might cause dysfunctional activation in language processing areas. Grounding upon the contribution of Hoffman et al. [20], the authors also hypothesized an increased activation or hypercoupling of speech production centers in the inferior frontal cortex and speech perception areas in left temporoparietal cortex. These areas seem to be specifically involved in the pathophysiology of AVHs, yet the model includes other brain regions such as Broca's area, the dorsolateral prefrontal cortex (DLPFC), orbitofrontal cortex (OFC), the supplementary motor area (SMA), the dorsal and ventral anterior cingulate (DaC; VaC), the cerebellum, and a number of subcortical regions (Figure 1).

Recent contributions are divided into “state” studies (which investigated brain areas involved during AVH experience) and “trait” studies (which investigate the tendency to hallucinate) [21]. State studies highlighted the role of areas related to inner speech production such as the bilateral inferior frontal gyrus (IFG) and the bilateral postcentral gyrus, plus the left inferior parietal lobe (IPL) that could play a role in the misattribution of the speech as not being self-generated. On the other hand, trait studies focused on areas associated with speech perception such as the left superior temporal gyrus (STG) and left middle temporal gyrus (MTG) and anterior cingulate cortex (ACC) that could similar to left IPL play a role in the distorted self-monitoring of speech [22]. An involvement is also shown for putamen and Wernicke's areas and its right homologue [23].

In conclusion, studies suggest that the core mechanisms of AVHs involve a complex functional loop including several brain areas interconnected, rather than a single pathway [23].

## 4. Auditory Verbal Hallucinations (AVH) in Other Disorders

Though the high prevalence in schizophrenia (40%–80%; [24]), AVHs are not specific for the disorder and, likewise, occur in the 10%–15% of nonclinical subjects [25, 26]. In healthy subjects, the presence of AVHs is frequently associated with subclinical levels of other schizotypal phenomena, plus an increased family loading for psychosis and axis I disorders and a higher prevalence of childhood trauma, but these subjects do not meet the criteria for schizotypal, schizoid, or paranoid disorders [27]. Furthermore, a crucial difference between the emotional content of AVHs in healthy individuals and patients with schizophrenia has been observed [28, 29]: while in schizophrenia AVHs are characterized by a negative emotional content leading to perceived distress and to a lower global functioning, voices of the healthy subjects mainly have a positive emotional content and are often not disturbing. This difference in the emotional content could distinguish between benign hallucinations and psychopathology.

In regard to clinical conditions, AVHs are frequently found in neurological or neurodegenerative disorders such as Alzheimer's disease, Parkinson's disease, dementia, hearing impairment and epilepsy [13, 30, 31], substance abuse [32], and in psychiatric conditions such as dissociative disorders, mood disorders, and borderline personality disorder [14, 33, 34]. In particular focusing on dementia, the presence of behavioral symptoms (mostly delusions and hallucinations) is significant, in particular in Alzheimer's disease and vascular dementia [35]: specifically, the prevalence of AVHs ranges between 1% and 29% [13]. Hallucinations seem to slightly increase correlating with the severity of dementia on the Clinical Dementia Rating [35], with greater percentage (21%) in moderate dementia. Findings also showed that psychotic symptoms in dementia frequently lead to caregiver distress and, consequently, to institutionalization and greater cognitive deterioration [13, 14]. 

## 5. Case Presentation

A seventy-six-years-old woman presented with her daughter to the Institute, complaining of hearing disturbing voices (AVHs according to Neuropsychiatric Inventory Clinician (NPI-C; [36]) started on August 2012. She reported she firstly suffered from hypnagogic hallucinations represented by religious choirs which later turned into hearing her dead newborn son's voice. These AVHs initially had a positive content, while afterwards she began to hear threatening voices associated with sleep disturbances. On presentation, she also reported fear of the dark and being left alone at home. A partial awareness of the hallucinating origin of the voices, typical in hallucinosis, distinguished her clinical picture from AVHs in psychosis. Her insight could be assessed through the Schedule for the Assessment of Insight (SAI; [37]): the woman showed quite high insight with regard to the recognition of her abnormal mental events and actively searched for treatment, accepting therapy with good adherence.

Magnetic resonance images showed bilaterally altered areas characterizing the periventricular and subcortical supratentorial white matter, in conjunction with vascular-degenerative subcortical encephalopathy features. The woman's cognitive clinical picture was a Mild Cognitive Impairment, though the long-term duration and the clinical evidence of progression set the diagnosis of dementia.

The pharmacological treatment essentially consisted of risperidone, gradually decreased. However, AVHs has been shown to be particularly difficult-to-treat and resistant to pharmacotherapy, suggesting that the improvement reached was not attributable to pharmacological treatment. 

On presentation, her cognitive function was assessed through the Mini Mental State Examination (MMSE; [38]) reaching a score of 25.24; the Symptoms Check-List 90-R (SCL 90-R; [39]) was also administered, showing high values on “obsessivity compulsivity” (40%), “depression” (37%), and “psychoticism” (50%) areas. The intensity and features of the auditory verbal hallucinations were assessed during a psychiatric interview, with the aid of the Neuropsychiatric Inventory Clinician (NPI-C; [36]). This instrument represents a comprehensive assessment tool for neuropsychiatric symptoms and psychopathology in Alzheimer's disease and other neurodegenerative disorders. The main results showed neither delusions nor agitation, irritability, aggression, disinhibition or aberrant motor and verbal disturbances. Conversely, as previously mentioned, the patient complained about daily threatening voices which caused her sleep disturbances and, consequently, significant distress. 

The woman was submitted to a 10 Hz rTMS treatment over T3P3 (temporoparietal cortex) at 80% motor threshold. The protocol of administration consisted in a 15-day daily treatment, with a stimulation sequence of 13 trains of 200 stimuli each (50 seconds intertrain interval), for an amount of 2600 stimuli. After treatment, the patient reported a marked reduction in AVHs: their frequency drastically decreased, their content loss threatening features, and, consequently, the associated distress considerably lowered. Also, no side effects were reported during all treatment and in the aftermath. 

## 6. Discussion

Previous studies regarding the use of TMS in the treatment of AVHs mostly employed the low-frequency rTMS protocol firstly proposed by Hoffman and colleagues [4]. Yet, to date, most literature concerning the employ of TMS in AVHs focused for the most part on schizophrenic patients, which have considerably different traits compared to elderly people with neurodegenerative disorders. In addition, the absence of delusional features in hallucinations set the patient's clinical picture markedly distant from psychosis: AVHs were not embedded in a delusional frame and not related to any particular delusional belief. Moreover, a partial awareness was preserved, almost giving the woman's AVHs the features of hallucinosis. This, in addition to neurodegenerative phenomena highlighted by MRI, explains our choice for a high frequency rTMS protocol. Given the evidence of links interruptions between strategic temporal and frontal brain regions [40], we hypothesized different features of AVHs between schizophrenia and dementia. In particular, we supposed the primary role of a disconnective syndrome rather than cortical hyperactivity [41–43] in dementia: this explains our choice of the rTMS protocol mentioned above. 

On the other hand, as far as we know, the literature concerning AVHs in elderly people did not contemplate the employ of TMS as a therapeutic tool. Hence, this contribution sets in a promising field of extending the therapeutic purposes of TMS: this latter proves as potentially useful also in some difficult-to-treat neurodegenerative disorders symptoms, such as behavioral symptoms. Currently, no specific therapeutic guidelines are available for behavioral symptoms, in particular for pharmacoresistant AVHs: TMS could represent a valuable treatment because of its effect on multiple symptoms. In particular, AVHs improvement is associated with mood and cognitive improvement: we actually cannot establish a causative connection affirming which is the primary target of rTMS treatment, but, as far as we know, we can observe its effects both on AVHs, mood, and cognition. Though further research is needed, this represents a first, encouraging result: next step could be an analysis of changes in functional and metabolic brain activity after rTMS treatment. 

## Figures and Tables

**Table 1:** Previous rTMS for AVHs studies.

Study	*N* active	*N* sham	Stimulated Area	Frequency	% MT	Session
Hoffman et al. [44]	12	12	T3P3	1 Hz	80	4
McIntosh et al. [45]	16	16	T3P3	1 Hz	80	4
Schönfeldt-Lecuona et al. [46]	11	10	STG	1 Hz	90	5
Chibbaro et al. [47]	8	8	T3P3	1 Hz	90	4
Fitzgerald et al. [48]	17	15	T3P3	1 Hz	90	10
Hoffman et al. [49]	27	23	T3P3	1 Hz	90	10
Lee et al. [50]	13	14	T3P3	1 Hz	100	10
Lee et al. [50]	12	14	T4P4	1 Hz	100	10
Poulet et al. [51]	10	10	T3P3	1 Hz	90	5
Brunelin et al. [52]	14	10	T3P3	1 Hz	90	10
Jandl et al. [53]	16	16	T3P3 T4P4	1 Hz	100	5
Jandl et al. [53]	16	16	T4P4	1 Hz	100	5
Rosa et al. [54]	6	5	T3P3	1 Hz	90	10
Saba et al. [55]	8	8	T3P3	1 Hz	80	10
Vercammen et al. [56]	16	16	T3P3	1 Hz	90	12
Vercammen et al. [56]	14	16	T3P3 T4P4	1 Hz	90	12
Loo et al. [57]	18	18	T3P3	1 Hz	110	3
Loo et al. [57]	18	18	T4P4	1 Hz	110	3
Slotema et al. [58]	22	20	T3P3	1 Hz	90	15
Slotema et al. [59]	20	20	fMRI	1 Hz	90	15

MT: motor threshold; T3P3: left temporoparietal cortex; T4P4: right temporoparietal cortex; STG: superior temporal gyrus; fMRI: functional magnetic resonance imaging.
